# Engineered multifunctional nanocarriers for controlled drug delivery in tumor immunotherapy

**DOI:** 10.3389/fonc.2022.1042125

**Published:** 2022-10-21

**Authors:** Theodora Katopodi, Savvas Petanidis, Drosos Tsavlis, Doxakis Anestakis, Charalampos Charalampidis, Ioanna Chatziprodromidou, Panagiotis Eskitzis, Paul Zarogoulidis, Christoforos Kosmidis, Dimitris Matthaios, Konstantinos Porpodis

**Affiliations:** ^1^ Department of Medicine, Laboratory of Medical Biology and Genetics, Aristotle University of Thessaloniki, Thessaloniki, Greece; ^2^ Department of Medicine, Laboratory of Experimental Physiology, Aristotle University of Thessaloniki, Thessaloniki, Greece; ^3^ Department of Histology, Medical School, University of Cyprus, Nicosia, Cyprus; ^4^ Department of Public Health, Medical School, University of Patras, Patra, Greece; ^5^ Department of Obstetrics, University of Western Macedonia, Kozani, Greece; ^6^ Third Department of Surgery, “AHEPA“ University Hospital, Aristotle University of Thessaloniki, Thessaloniki, Greece; ^7^ Oncology Department, General Hospital of Rhodes, Rhodes, Greece; ^8^ Pulmonary Department-Oncology Unit, “G. Papanikolaou” General Hospital, Aristotle University of Thessaloniki, Thessaloniki, Greece

**Keywords:** nanocarriers, nanogel, microneedles, nanodrugs, immunotherapy

## Abstract

The appearance of chemoresistance in cancer is a major issue. The main barriers to conventional tumor chemotherapy are undesirable toxic effects and multidrug resistance. Cancer nanotherapeutics were developed to get around the drawbacks of conventional chemotherapy. Through clinical evaluation of thoughtfully developed nano delivery systems, cancer nanotherapeutics have recently offered unmatched potential to comprehend and combat drug resistance and toxicity. In different design approaches, including passive targeting, active targeting, nanomedicine, and multimodal nanomedicine combination therapy, were successful in treating cancer in this situation. Even though cancer nanotherapy has achieved considerable technological development, tumor biology complexity and heterogeneity and a lack of full knowledge of nano-bio interactions remain important hurdles to future clinical translation and commercialization. The recent developments and advancements in cancer nanotherapeutics utilizing a wide variety of nanomaterial-based platforms to overcome cancer treatment resistance are covered in this article. Additionally, an evaluation of different nanotherapeutics-based approaches to cancer treatment, such as tumor microenvironment targeted techniques, sophisticated delivery methods for the precise targeting of cancer stem cells, as well as an update on clinical studies are discussed. Lastly, the potential for cancer nanotherapeutics to overcome tumor relapse and the therapeutic effects and targeted efficacies of modern nanosystems are analyzed.

## Introduction

During the last decade, cancer immunotherapy is a promising tool in the battle against cancer. Tumor immunotherapy, in contrast to conventional treatments, primarily targets immune cells. It stimulates the body’s immune response by preventing harmful immune regulatory factors and improving immune cells’ capacity to identify tumor cell surface antigens and eradicate tumor cells (1). According to reports, immunotherapy has a number of benefits, including improved effectiveness, less side effects, and the ability to inhibit tumor relapse (2). Numerous new immunotherapies have been created in quick succession in recent years thanks to a deep understanding of the processes by which tumors elude the immune system (3). However, clinical applications of cancer immunotherapy are still very limited and are primary based on T cell-mediated immunity (4). The main disadvantages of immunotherapy regimen include low patient response rates and severe immune-related adverse effects (5, 6). This drawback can be attributed to the high tumor heterogeneity and immunosuppressive tumor microenvironment which amplify tumor burden (7, 8). Nanotechnology provides an alternative strategy for tumor-targeted therapy. In the last years, recent advances in materials science and nano-biotechnology have revolutionized drug development and research (9, 10). Tumor nanotherapy presents new opportunities against cancer-related drug resistance and tumor recurrence. Especially, recent nanotherapeutics-based approaches can overcome the restraints of conventional tumor immunotherapies (11–13). Since the production of the first tumor immunotherapy drug (IFN-a) and the latest immunotherapy regimes like checkpoint inhibitors (PD-L1, CTLA-4), CAR T cell therapy, tumor vaccines and oncolytic viruses, nanotherapy has become a new exemplar for clinical cancer treatment (14–16). In this review, the recent progress of nanomedicine engineering for combinational tumor-targeted immunotherapy is summarized with emphasis in existing challenges, and future perspectives in the field.

## Nanodrug-targeted tumor immunotherapy

**Figure 1:**
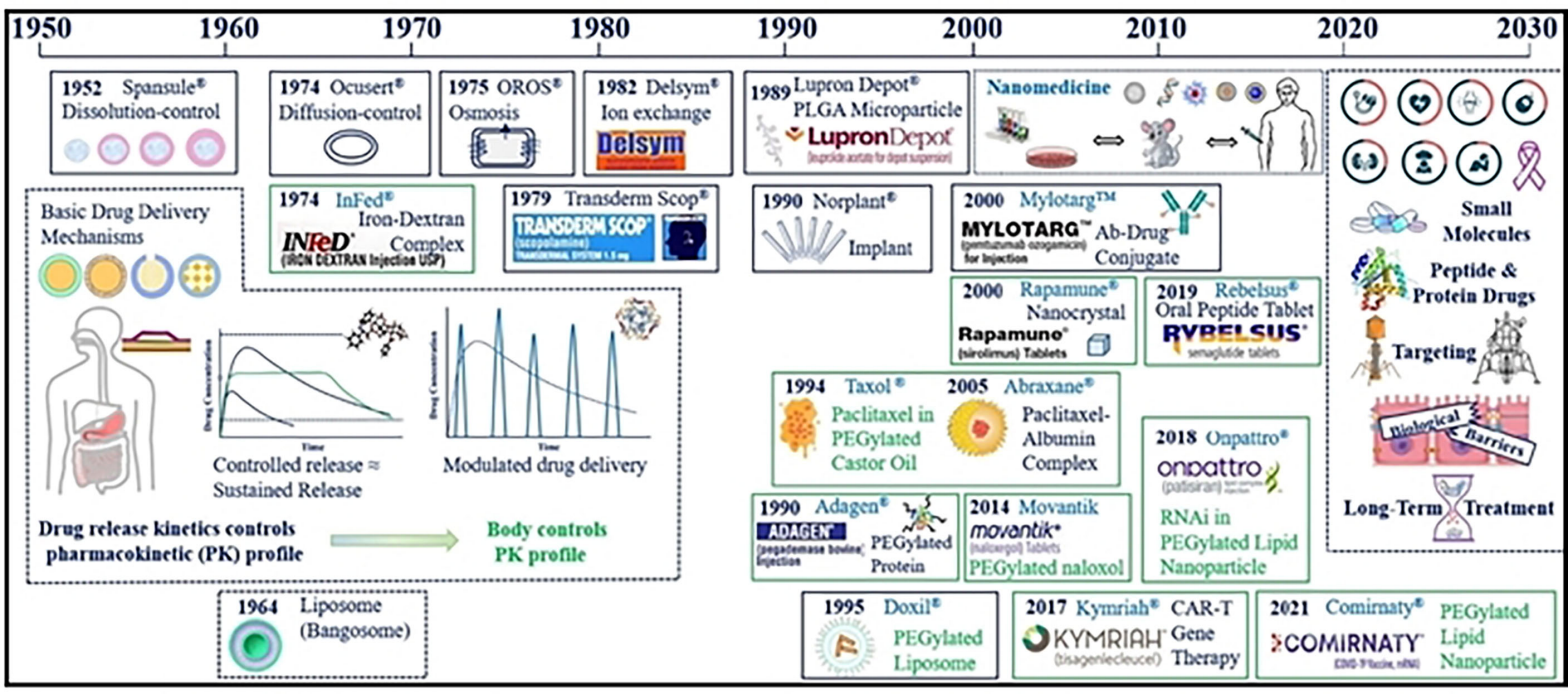
Major advances in cancer nanotherapeutics and drug delivery systems. Reproduced with permission from Ref (11). Copyright 2022, Elsevier.

Compared with the traditional chemo/immunotherapy scheme, modern nanocarriers display several advantages like increased bioavailability, lower toxicity, improved solubility and enhanced targeting (17, 18). Since the early discovery of liposomes in 1961, Doxorubicin was the first liposome nanodrug to be approved by FDA in 1994 (19) (**Figure 1**). Currently numerous nanodrugs are under clinical trials for diagnosis, treatment, and prevention of tumors as well as autoimmune or neurodegenerative diseases (20, 21). Both enhanced drug targeting and increased drug efficacy of nanodrugs can improve drug accumulation at the tumor site due to their unique high permeability and long retention (EPR) effect (22–24). The EPR effect has been recognized as a limitation in clinical tumors. In fact, several strategies directed to assist the accumulation of nanocarriers beyond the levels of EPR effect, which can promote the efficacy of immunotherapeutics are being considered (25). Failure of chemo/immunotherapy due to difficulty in penetrating the blood–brain barrier (BBB) and low therapeutic index are also critical issues for therapeutic regimens (26, 27). In addition, drug resistance due to tumor heterogeneity, and the immunosuppressive hypoxic tumor microenvironment impacts patients’ quality of life and creates a huge healthcare burden (28, 29). Hence, to overcome these limitations of conventional chemotherapy novel nanotherapeutic platforms were employed for efficient cancer therapy (30–32).

## Nanotherapy in tumor targeting

**Figure 2:**
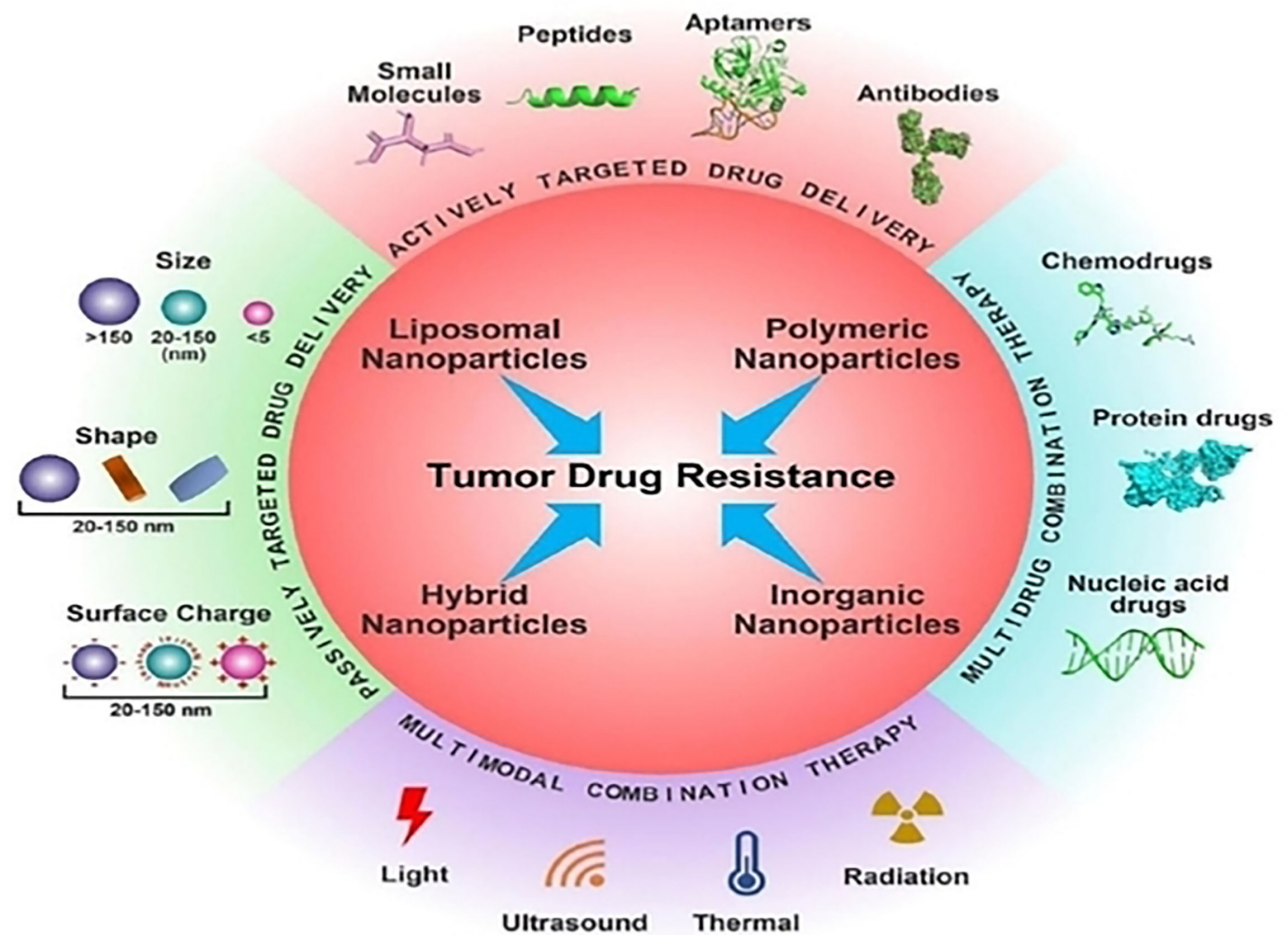
Several cancer nanotherapeutic strategies are employed for the efficient targeting of tumor cells. Reproduced with permission from Ref (29). Copyright 2022, Elsevier.

Cancer nanotherapeutics can be employed for the efficient targeting of tumor cells enabling passive targeted drug delivery (**Figure 2**) (33, 34). Furthermore, the functional or structural modification of the nanocarriers can reduce the off target rate and enhance drug circulation time, improve drug stability and solubility (35, 36). In addition to cancer treatments, drugs based on nanotechnology have important potential implications for imaging diagnostics and prognosis of many tumors that are drug-resistant (37, 38). By targeting the tumor microenvironment (39), nanotherapeutics, have exhibited great potential in recent years due to the use of siRNA/miRNA interference techniques (10), exosome-based delivery systems and self-assembly prodrug (SAP) method. In addition, nano therapies can also target specific components of the tumor microenvironment like integrins and CSCs, especially in chemoresistant relapsed tumors (40, 41).

## Controlled drug delivery

To accomplish the intended treatment outcome, controlled drug delivery systems (DDS) distribute therapeutic compounds to specified target cells, tissues, or organs in a pre-designed and controllable manner (35). Traditional drugs have limited intestinal permeability, low solubility, and poor absorption; as a result, they have low bioavailability and considerable variability. In nanotherapeutics, researchers use hydrogels and nanoparticles to achieve their goals because proteins and peptides frequently have a short half-life in serum (42). Controlled drug release can be attained by actively targeting particular locations, altering drug release kinetics, and/or inducing drug release in physiological conditions that are specific to different tissues (43). Conventional DDSs have significant disadvantages, such as systemic application and the possibility that they degrade or become inactive before acting, which could lead to an inadequate dose reaching the target site (44). Traditional TDDSs (transdermal DDSs) may be regarded as targeted because they can be applied close to the intended site of action, but their pharmacological behaviors are not precisely self-controlled due to low skin permeability or their inability to penetrate the (SC) stratum corneum, for example, by hydrophilic drugs, uncontrolled drug diffusion, and non-specific targeting. Additionally, depending on their physicochemical qualities, any added excipients, the structure and thickness of the skin, the delivery efficiency, or the penetration and absorption of active pharmaceutical ingredients (APIs), is frequently limited (45). Because they significantly increase the effectiveness and control of drug delivery, MN-based controlled TDDS are able to overcome these problems. The ease of self-administration and painlessness of MN-systems can provide exact localization of the drug with reduced dose frequency, which increases patient compliance. Thus, they can be utilized to keep constant the drug concentrations in tissues, blood, and particular target areas (46).

## Targeting the TME

**Figure 3:**
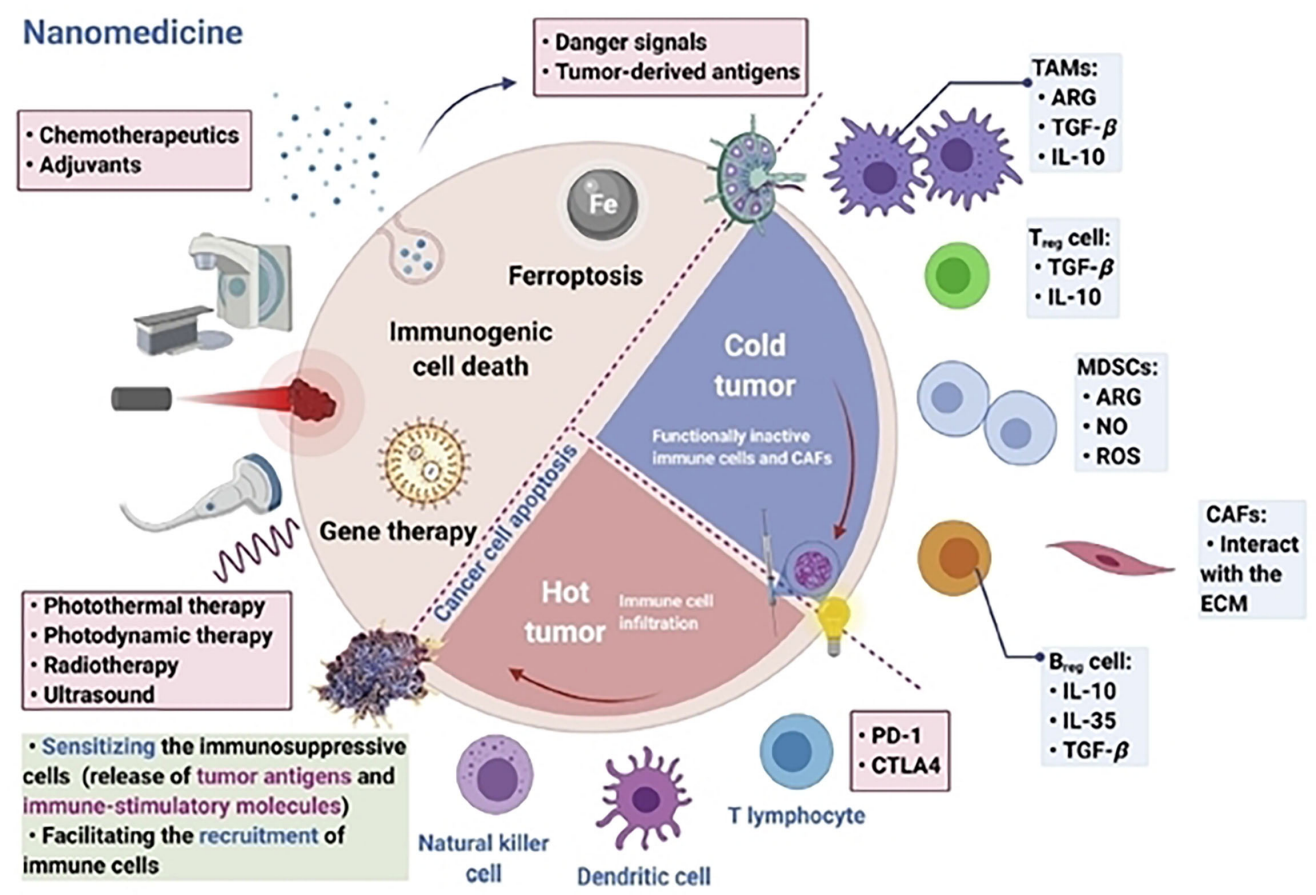
Targeting immunosuppressive cells inside the tumor microenvironment is vital for modern nanomedicine-based immunotherapy. Reproduced with permission from Ref (55). Copyright 2022, Elsevier.

The tumor microenvironment (TME) is a key factor in the development of tumor heterogeneity and disease pathology (47, 48). Drugs cannot penetrate the tumor tissue due to the heterogeneity of TME and its constituent cells, interstitial fluid, and ECM, which function as physical barriers (49, 50). As a result, distinct gradients in drug concentrations and cell proliferation can affect the sensitivity of tumor cells to treatment (51). Drugs used for cancer treatment are resistant to this mechanism. Multiple drug resistance (MDR) offers significant unresolved issues in cancer chemotherapy, and roughly up to 70% of patients experience tumor relapse issues as a result of MDR (52). Through a number of mechanisms, including cell-cell and cell-ECM interactions, crosstalk between various cell types, phenotypic alterations, mechanosensing variation, and protective dormancy, TME and its components trigger and promote drug resistance (53, 54). Furthermore, infiltration of immunosuppressive cells inside the TME (**Figure 3**), like cancer-associated fibroblasts, MDSCs, cancer-associated vascular endothelial cells, pericytes, lymphatic endothelial cells (LECs), and CSCs supports the heterogeneity of the TME (56). In addition hypoxia, that creates an acidic environment, the extracellular matrix, cytokines, growth hormones, and vascular networks establish prolonged immunosuppression inside the TME. These key components of the TME foster a favorable and permissive environment for the growth of cancer cells (57). For example, leaky vasculature, insufficient vascular perfusion, an acidic environment, abnormal pH dynamics, altered enzyme expression, altered metabolism, and hypoxic conditions are just a few of the traits that TME employs to set up the tumorigenic mechanism (58).

## Photothermal therapy

**Figure 4:**
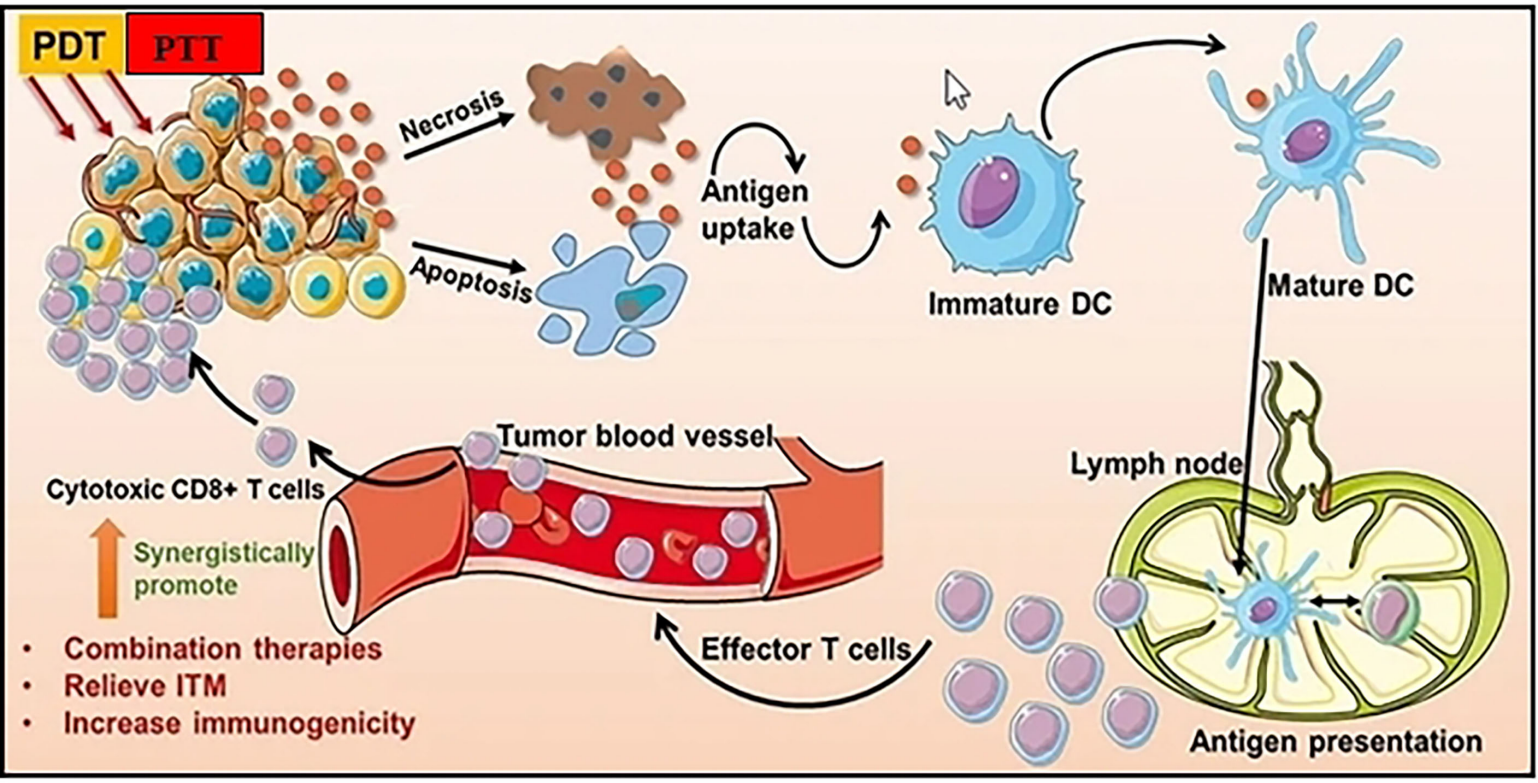
Photothermal and Photodynamic therapy can improve antitumor immune responses and increase tumor immunogenicity. Adapted with permission from Ref (59). Copyright 2021, Dove Press.

In photothermal therapy (PTT), cancer cells are killed by the created heat of exposing photothermal agents to light. Due to its noninvasiveness, high spatiotemporal precision, ease of usage, and flexibility of light sources, PTT has demonstrated considerable potential for the treatment of cancer (**Figure 4**) (60, 61). As a result, PTT frequently exhibits remarkable selectivity and specificity for malignant cells without obviously harming neighboring healthy tissues. It is well known that nanoparticles exhibit distinctive magnetic and optical properties. These characteristics have led to the observation that nanoparticles can increase the temperature of cancer cells by absorbing near-infrared light (NIR), electromagnetic waves, and radiofrequency waves (RW) (62). Nanoparticles have the ability to remotely and locally heat tumor cells. Due to their tiny size, biocompatibility, dispersibility in biocompatible solvent, bioavailability, and ability to generate heat when activated externally, nanoparticles are advantageous for thermal treatments in biological systems (63). Thermal therapeutic applications have been reported to use a variety of nanoparticle formulations. Broadly speaking, they can be divided into magnetic and non-magnetic nanoparticles. The majority of PTT nanodrugs absorb light in the visible (400-700 nm) or near-infrared (NIR) (700-1,350 nm) ranges. Diode lasers (630-1,100 nm), dye lasers (390-1,000 nm), alexandrite lasers (720-800 nm), and neodymium-doped yttrium aluminum garnet (Nd : YAG) lasers (1,064 nm) are suitable lasers that may excite these substances.

## Immunogenic cell death

Immunogenic cell death (ICD), also known as immunogenic apoptosis, is a type of cell death that triggers a controlled immunological response. ICD promotes antitumor immune signaling against a variety of solid tumors in addition to directly killing tumor cells. Such methods for producing vaccine-like properties could be utilized to encourage an immunogenic, “hot” tumor microenvironment to develop from a “cold” tumor milieu, increasing patient response rates and producing positive therapeutic effects. ICD is characterized by the release of TAAs, DAMPs, and pro-inflammatory cytokines that make it easier for TAAs to be presented to adaptive immune cells and trigger an immunological reaction specific to an antigen. For example, in syngeneic animal models, Doxil enhances antitumor responses by cooperating with cancer immunotherapies (64). When combined with anti-PD-L1, doxil therapy enhanced the percentage of CD8^+^T cells that infiltrated tumors while decreasing the percentage of regulatory T cells Doxil treatment boosted mature dendritic cells’ expression of CD80 in the tumor indicating that Doxil may cause these tumor-infiltrating cells to develop a costimulatory phenotype that can stimulate an anticancer T-cell response. Recently, PTEN-negative glioblastoma (GBM) is eradicated with anti-PD1 therapy assisted by Epirubicin-loaded nanosomes. By converting cold GBM into hot tumors with high infiltration of antitumor immune cells through the induction of ICD, removal of immunosuppressive MSDCs, and reduction of PD-L1 expression on tumor cells, the combination of epirubicin-loaded micelles with aPD1 eliminated GBM resistance to ICI (65).

## CSC-based nanotherapy

Tumor heterogeneity is a major barrier to cancer therapy because the majority of tumors contain different cell types that respond differently to chemotherapy (66). CSCs, which regulate the tumor microenvironment and exhibit self-renewal ability, invasiveness, and high tumorigenicity, are one of the critical factors responsible for tumor heterogeneity (67). In recent years, prospective nanotherapeutic strategies for targeting CSCs have included critical factors required for CSC survival in the tumor microenvironment, such as specific surface biomarkers (ALDH, CD44, CD133, EpCAM), drug efflux pumps like the ABC transporters, various metabolic routes, and stem cell signaling pathways (Hedgehog, Notch, Wnt). Lately, engineered novel nanoparticles co-loaded with retinoic acid and the chemotherapeutic drug camptothecin could efficiently overcome the chemotherapeutic resistance of CSCs. When all-trans retinoic acid is released, leads to CSC differentiation in the hypoxic TME. This dual strategy allows for controlled drug release in CSCs while also reduces stemness-related drug resistance, improves chemotherapeutic response and prevents post-surgical tumor relapse and metastasis in breast tumor mouse models (68). In addition, doxorubicin-encapsulated polymeric nanoparticle surface-decorated with chitosan can specifically target the CD44 receptors of tumor reinitiating cancer stem-like cells which are responsible for cancer recurrence. This nano design strategy increases the cytotoxicity of the doxorubicin by six times in comparison to the use of free doxorubicin and eliminates CD44^+^ cancer stem-like cells residing in 3D mammary tumor spheroids (69). Another research group, used riboflavin-loaded intracellular vesicles coated with the ATP binding cassette ABCG2 to specifically target CSCs and found a higher accumulation of riboflavin within the cytoplasm due to specific recognition properties (70). An additional study used pH responsive/hypoxia responsive riboflavin linked to three-pronged nanoparticles to target both tumor cells and CSCs. These nanosomes can efficiently eliminate differentiated cancer cells, CSCs, and vascular niches for synergistic inoperable tumor therapy. In order to kill those three cell types simultaneously, drugs irinotecan (Ir), cyclopamine (CP), and erlotinib (ET) targeting differentiated cancer cells, CSCs, and endothelial cells of the vascular niches are used (71).

## Micro-RNA targeting

During the last decade, RNA-based therapeutics represent an attractive approach for the treatment of cancers, as well as many other diseases and support approaching ‘‘undruggable’’ targets (72). As emerging gene regulators, miRNA-based cancer therapeutics have enormous implications in cancer pathophysiology. MiRNAs are small, noncoding RNAs that control gene expression from cell growth and differentiation to apoptosis, and tissue development (73). Deregulation of miRNAs expression can trigger cellular transformation, altered metabolism, and carcinogenesis (74). MiRNAs’ differential expression in tumor-related tissues allows them to target a wide range of transcripts involved in cancer signaling pathways. Due to their dual roles as tumor suppressors and oncogenic miRNAs, miRNAs have the ability to modify a number of signaling pathways involved in cancer and metastasis *via* the transcriptional effect (75). As a result, miRNAs can be targeted in cancer therapies either as artificial anti-miR sequences for miRNAs that are upregulated or as miRNA mimics for miRNAs that are downregulated. In this situation, miRNAs may be repressed to activate tumor suppressor genes or weaken genes that prevent apoptosis (76). Recently, a nano formulation was created that uses biodegradable porous silicon nanoparticles (pSiNPs) to encapsulate an anti-miR-21 locked nucleic acid payload and display a tumor-homing peptide for targeted delivery in order to create an enhanced anti-miR therapeutic agent for the treatment of ovarian cancer (77). On a variety of ovarian cancer cell lines, targeting effectiveness, miR-21 silencing, and anticancer activity are all optimized *in vitro*. A formulation of anti-miR-21 in a pSiNP exhibiting the targeting peptide CGKRK is used for *in vivo* evaluation. When this nanoparticulate material is administered in tumor xenograft mice, the silencing of miR-21 results in a significant suppression of tumor growth. The therapy of ovarian cancer in a mouse xenograft using tumor-targeted anti-miR porous silicon nanoparticles is demonstrated in this study. Researchers have developed developed a peptide-based surface-fill hydrogel (SFH) that can be injected or sprayed directly onto surface tumors after surgery or as a primary therapy. Once used, SFH has the ability to adjust shape in response to changes in tissue morphology that might take place during surgery. Nanoparticles made of microRNA (miRNA-215 and miRNA-206) and intrinsically disordered peptides are released by implanted SFH and infiltrate cancer cells to lessen their oncogenic hallmark. Four preclinical models of mesothelioma respond to a single application of SFH, suggesting the therapeutic value of local tumor-specific microRNA therapy (78).

## Exosome signaling

**Figure 5:**
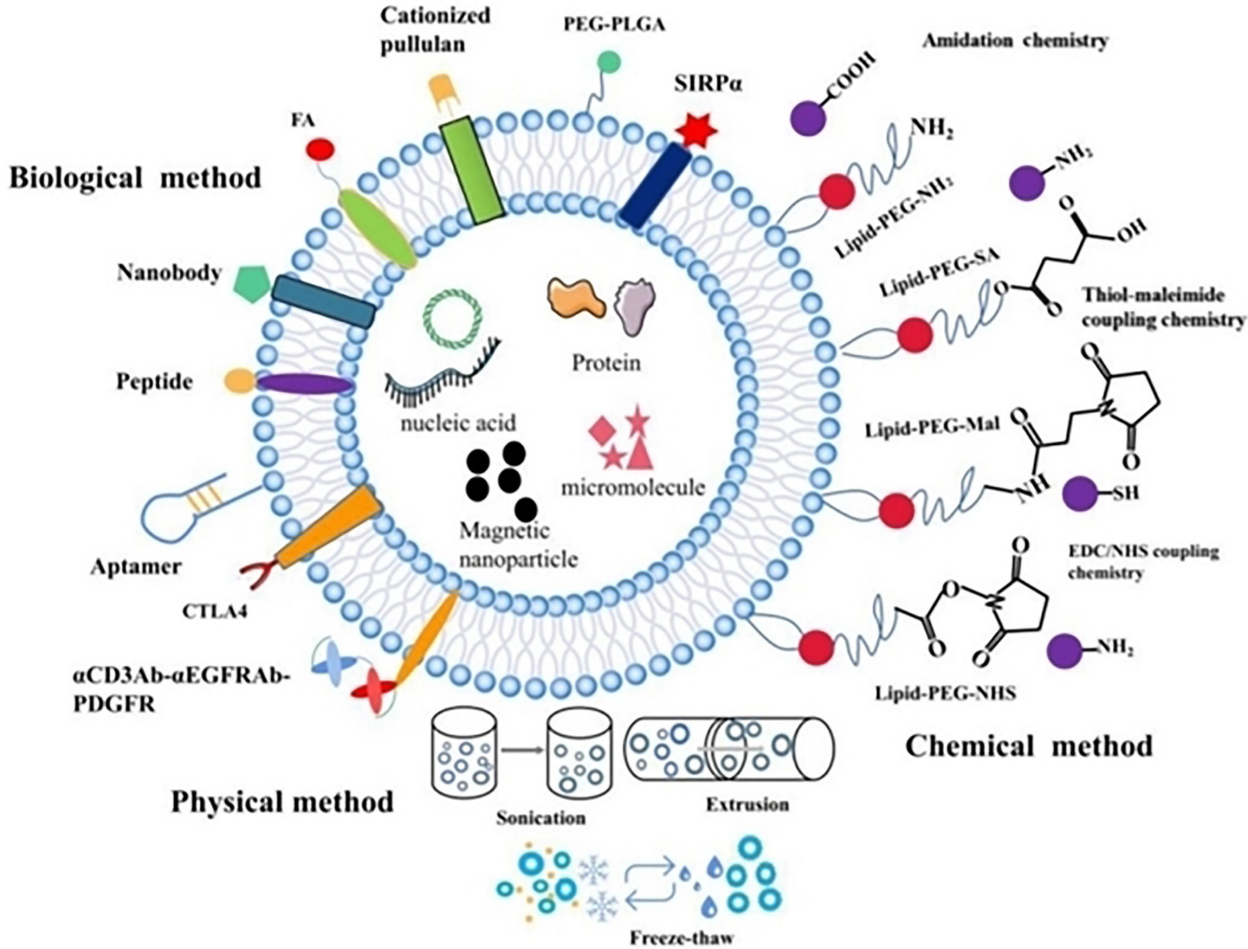
Exosomal surface engineering for precision targeting in cancer nanotherapeutics. Reproduced with permission from Ref (86). Copyright 2021, Ivyspring.

Exosomes are a subclass of cell-derived extracellular vesicles (EVs) with an average diameter of 30-150 nm that are released by a wide range of cells throughout the body (79, 80). They are secreted by a variety of cells found in the tumor microenvironment, including cancer cells, tumor associated fibroblasts, CSCs, and tumor associated immune cells (81). Exosome-mediated continual interaction between tumor cells and stromal cells constitutes a significant portion of the communication in the tumor microenvironment (82). Exosomes play a role in a number of cellular and pathological situations and convey their payload to nearby tissues as well as distant organs *via* intercellular communication (83). Proteins, lipids, nucleic acids, and metabolites make up the exosome cargo, which also controls immune response, stimulates angiogenesis, and modifies cancer-related signaling pathways in the tumor microenvironment. Exosomes have a crucial role in conferring drug resistance to cancer cells *via* intercellular interactions in a range of cancer types, according to numerous *in vitro* and preclinical *in vivo* investigations (84).Through the control of drug efflux and metabolism, epithelial-mesenchymal transition, modification of prosurvival signaling pathways, remodeling of the tumor microenvironment, and increased concentration of plastic CSCs, the exosome cargo mediates chemoresistance (85). Exosomes have a critical role in the development of drug resistance in cancer, as well as in the dissemination of drug resistance phenotypes to other cancer cells. Exosomes may be employed as a therapeutic target to treat cancer cells with drug resistance because of their role in chemoresistance (**Figure 5**) (87). Exosomes can serve as desirable nanocarriers for the delivery of specific drugs or genes. By modifying exosomes with tumor-specific peptides, proteins, or antibodies for precise targeted drug delivery, their specificity may be increased still further. The development of an effective cargo loading technique and selection of exosome-producing cells are crucial steps in using exosomes as nanocarriers because they have a significant impact on the function, biodistribution, and immunogenicity of the exosomes. In a recent study, exosome-based delivery of paclitaxel to MDR cancer cells was successfully achieved with overexpression of efflux transporters P-glycoprotein (P-gp). In a lung cancer xenograft model, paclitaxel-loaded exosomes demonstrated the reversal of drug resistance by increasing sensitivity to MDR cancer cells, evading P-gp-mediated drug efflux, and suppressing metastasis (88). Furthermore, by combining the chemoresistance drug doxorubicin with entire monocyte or macrophage cells and then passing the mixture through filters with various pore diameters, Jang et al. created bioinspired exosome-mimetic nanovesicles. Comparing the created exosome mimics to doxorubicin-loaded natural exosomes revealed similar characteristics but a 100-fold higher production yield (89). Exosomes from bone marrow mesenchymal stem cells were used by Wu et al. (2020) to target leucine rich repeats and showed decreased cisplatin resistance in NSCLC (90). In a different investigation, synthetic exosomes were used in conjunction with the miR-21 inhibitor 5-FU to reverse drug resistance in colon cancer through targeted chemotherapy (91). Finally, exosomes loaded with RAD51 siRNA were shown by Shtam et al. to diminish DNA damage-repair protein levels and induce apoptosis in cervical cancer and fibrosarcoma cell lines.

## Self-assembly prodrug

For administering poorly soluble anticancer drugs, self-assembling prodrugs (SAPs) constitute a reliable and potent nanotherapeutic strategy. The maximal drug loading capacity, regulated drug release kinetics, longer blood circulation, and preferential tumor accumulation based on the EPR effect are only a few of the many inherent benefits of SAPs (92). These prodrug conjugates enable effective self-assembly into nanodrugs with the capacity to encapsulate other therapeutic agents with distinct molecular targets, enabling concurrent temporal-spatial drug release for synergistic anticancer efficacy with less systemic side effects. The SAP technique has gained significant attention over the past 20 years as a potent therapeutic platform for the improvement of targeted tumor treatment (93, 94). Drug-drug conjugates, polymer-drugs, and lipid-drugs are the three categories into which SAPs nanotherapeutics are divided [270]. Due to its simplicity in formulation, high hydrophilicity, and biocompatibility, hydrophilic polyethylene glycol (PEG) was frequently employed in earlier studies in combination with lipophilic drugs in order to circumvent the solubility and bioavailability issues associated with free pharmaceuticals (95). In addition to self-assembling into various nano formulations, such as polymeric micelles, PEG-based prodrugs also co-deliver the water-insoluble chemotherapeutics included in their hydrophobic core, which results in synergistic anti-cancer efficacy (96). Recently, a study demonstrated that platinum Pt (IV) prodrugs based on cisplatin and chemosensitizer adjudin (ADD), which had the capacity to self-assemble into nanosheets, improved the sensitivity of cisplatin to triple-negative breast cancer. With a 266-fold reduced IC50 value, these Pt (IV)-ADD-based self-assembled prodrug nanotherapeutics demonstrated superior *in vivo* tumor growth suppression (97). In addition, Domvri et al. demonstrated the use of PLGA-PEG biocompatible nanocarriers (NCs) for the precise targeting of MDSCs inside the lung tumor microenvironment. This is done by combining L-Norvaline and Sunitinib with biodegradable nanosomes which inhibit tumor-related immunosuppression (98). In a different study, self-assembling doxorubicin prodrug PEG2K-DOX showed superior *in vivo* therapeutic efficacy against MDR xenograft tumors to doxorubicin alone and the effective reversal of doxorubicin-related drug resistance (99). Likewise, Yang et al., showed that simple insertion of a trisulfide bond can turn doxorubicin homodimeric prodrugs into self-assembled dimeric nanodrug with increased drug loading, high self-assembly stability, and enhanced tumor selectivity (100). Recently, synthetic peptide-based rotor molecules could target the microtubule array and self-assemble in response to their environment. Such nano-bio interactions cause atypical prometaphase—metaphase oscillations that impede the proliferation of different cancer cells without obviously causing neurotoxicity. They do this by suppressing local tubulin polymerization. In the subcutaneous cervix cancer xenograft tumor model, the nanosomes also have powerful antiproliferative effects that are superior to Cisplatin and Taxol, the traditional antimitotic medication (101).

## Nanogels

**Figure 6:**
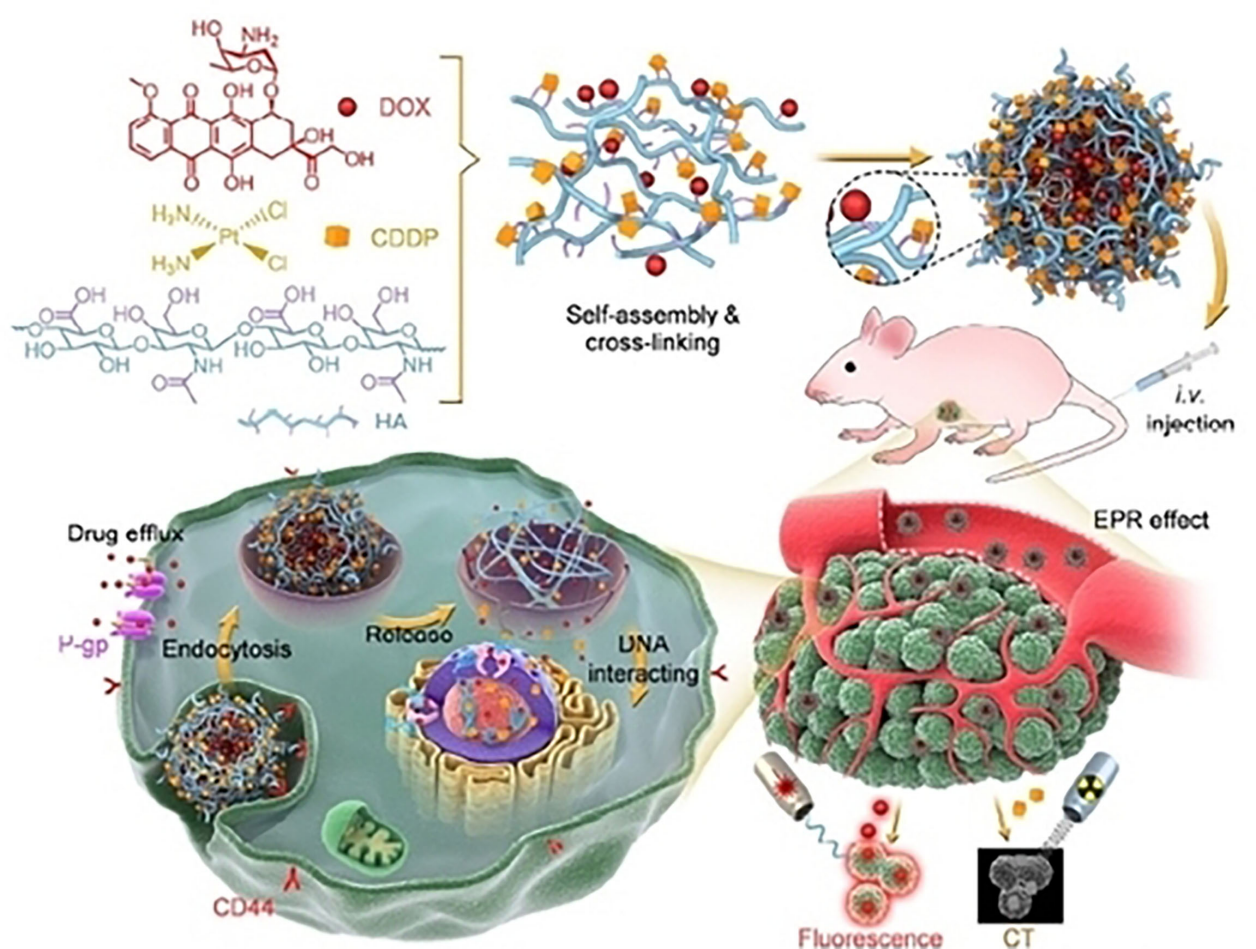
An example of hyaluronate nanogel for targeting multidrug-resistant and efficient delivery of doxorubicin and cisplatin in breast cancer. Reproduced with permission from Ref (103). Copyright 2021,Springer Nature.

Nanogels are hydrogels with a particle size between 20-250 nm and have a 3D permeable structure (102). Nanogels are a type of systemic drug delivery carriers and are made up of different natural polymers, synthetic polymers, or mixtures of both, which helps to encapsulate proteins, oligonucleotides, and small compounds. Due to their special characteristics, nanogels can be used for imaging, diagnostics, and drug delivery (**Figure 6**). The development of nanoplatforms based on nanogels holds great promise for the future of drug design. The ability to encapsulate hydrophilic or hydrophobic compounds, small-molecule chemicals and proteins, DNA/RNA sequences, and even ultrasmall nanoparticles, is exhibited by nanogels, made by chemical crosslinking or physically self-assembling. The carriers’ nanoscale structure gives them a precise surface area and interior volume, enhancing the stability of loaded drugs and extending their stay in circulation. It has been demonstrated that reactions or the breaking of chemical bonds within the structure of drug-loaded nanogels cause the regulated or sustained release of the drug. Recently, a Paclitaxel-based mucoadhesive nanogel with multivalent interactions for cervical cancer therapy was constructed. This nanogel can reverse multidrug resistance effectively, and successfully suppress tumor growth (104). In addition, to overcoming cisplatin resistance, a multifunctional nanogel was engineered that can promote apoptosis and reverse cisplatin resistance in lung cancer. This Valproate-D-Nanogel can induce G2/M arrest and trigger the intracellular apoptotic ROS-P53 pathway in cisplatin-resistant lung adenocarcinoma (105). Furthermore, engineered doxorubicin (DOX)-loaded polypeptide nanogel displays prolonged circulation and enhanced intratumoral accumulation in stage III NSCLC (106).

## Microneedles

**Figure 7:**
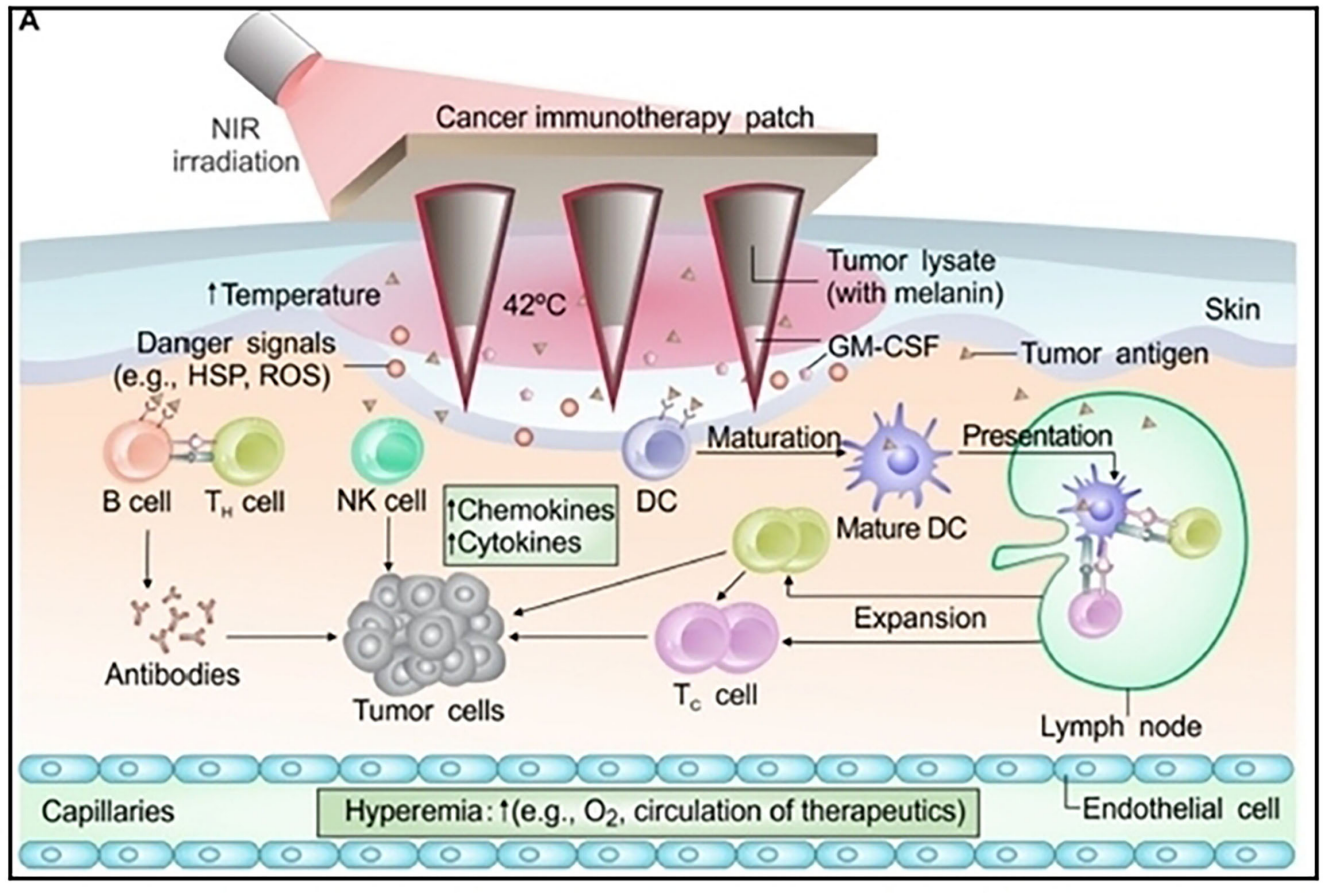
Schematic illustration of MN-based transdermal vaccination patch used for immunotherapy treatment of melanoma. Reproduced with permission from Ref (109). Copyright 2017, AAAS.

Drug delivery in the proper amount, at the proper time, and at the proper spot could considerably improve its efficacy in cancer treatment (107, 108). This can be accomplished by utilizing Microneedles (MNs), which are frequently seen as a synergistic combination of transdermal patches and hypodermic needles (**Figure 7**) (110). MNs create micro-channels in the epidermis of the skin that allow drugs to be delivered directly to the tumor site (111). In comparison to hypodermic needles and syringes, they are straightforward, non-invasive, and safer. MNs are preferable over hypodermic needles since they result in reduced or no pain at the administration location. Most importantly, site-specific administration aids in minimizing side effects and protecting the safety of chemotherapy patients by limiting the loss of healthy normal cells. Specifically, engineered microneedles can decrease the expression of heat shock proteins, reduce ATP levels and sensitize tumor cells to photothermal therapy. These glucose oxidase (GOx) and catalase (CAT) nanoreactors can significantly increase antitumor efficacy and display efficient tumor accumulation (112). Furthermore, an innovative self-degradable microneedle (MN) patch for the sustained delivery of aPD1 was used in melanoma treatment. In detail, a microneedle patch was engineered that encapsulates aPD1 and glucose oxidase (GOx), and triggers an immune response. The patch can convert blood glucose to gluconic acid thus creating an acidic environment which promotes the release of aPD1 in a B16F10 mouse melanoma model (113). Likewise, microneedles loaded with anti-PD-1 cisplatin nanoparticles were used for T-cell activation, boosted immune response, blockage of PD-1 in T-cells by aPD-1, and synergistic cancer immuno-chemotherapy (114). In a similar manner, dissolving microneedles can efficient deliver the R848 Toll-like receptor 7/8 agonist and hydrophilic antigens EG7-OVA to tumor-bearing mice and induce an antigen-specific immunity (115).

## Tumor antigen delivery

**Figure 8:**
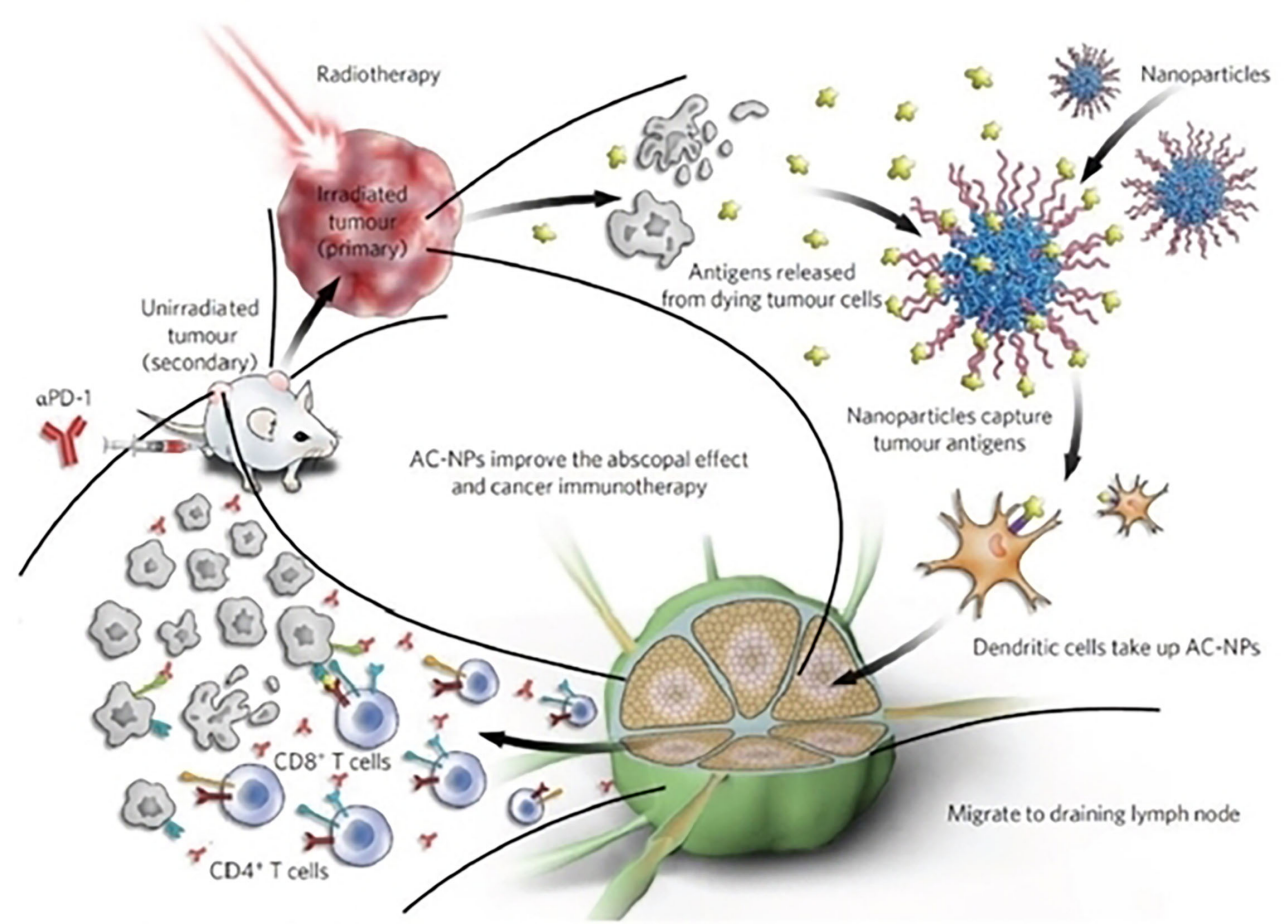
Schematic illustration of antigen-capture nanoparticles used for tumor immunotherapy. Reproduced with permission from Ref (116). Copyright 2017, Nature Publishing Group.

Nearly all tumor cells produce antigens or tumor-associated antigens (TAAs) that can be used in order to boost anticancer immune responses (69, 70). Nanoparticles can protect the antigen from degradation and deliver it directly to dendritic cells (DCs) or T-lymphocytes in order to propagate T-cell activation and response (**Figure 8**). This effectively stimulates cytotoxic T-lymphocytes (CTLs) and promotes anti-tumor immunity (117). Specifically, antigen-capturing nanoparticles (AC-NPs) can deliver tumor-specific proteins to APCs and significantly improve the efficacy of αPD-1 treatment and induce an expansion of CD8+ cytotoxic T cells and increased both CD4^+^T/Treg and CD8^+^T/Treg ratios (116). Also, targeted delivery of peptide antigens to tumor macrophages (TAMs) by nanogel can overcome tumor immune resistance and sensitizes the resistant tumors to T cell dependent immunotherapies (118, 119). Furthermore, doxorubicin (DOX) chemotherapy drug and oxygen are delivered by a hemoglobin- (Hb-PCL) conjugate self-assembled biomimetic nano red blood cell (nano-RBC) system for reprogramming of the hypoxic immunosuppressive TME. The Hb moiety of this nano system can preferentially target the M2-type TAMs *via* the CD163 surface receptor and bind to endogenous plasma haptoglobin (Hp), thereby killing tumor cells. Additionally, the O_2_ generated by the Hb reduces tumor hypoxia, which strengthens the immune response against the tumor by deterring the recruitment of M2-type macrophages. The TME is synergistically reprogrammed by TAM-targeting depletion and hypoxia relief, which simultaneously reduces tumor cell PD-L1 expression, decreases the levels of immunosuppressive cytokines like IL-10 and TGF-b, increases the expression of immunostimulatory IFN-γ, improves cytotoxic T lymphocyte (CTL) response, and triggers a strong memory response (120). Tumor antigens can also be loaded onto nanoerythrosomes which activate T cell immune responses and trigger high CD8 T cell infiltration and tumor regression in B16F10 and 4T1 tumor models (121).

## Conclusions

Cancer nanotherapeutics have demonstrated a promising therapeutic alternative to current chemotherapy/immunotherapy regimens for overcoming or reversing drug resistance. Before being employed in clinical trials, these agents need to be better characterized and optimized. A better understanding of the tumor microenvironment, and development of novel approaches (CSCs targeting, nucleic acids delivery, exosomal loading), and clinical trials utilizing nanosome-based systems are all suggested by the rapid development of nanotechnology and materials science. Utilizing nanotechnology’s advantages in tumor immunotherapy can successfully prevent drugs from unintentional deterioration and aid in achieving long-term circulation in both the blood and target tumor sites. The structure of nanoparticles needs to be changed in order to overcome the many issues restricting the present tumor immunotherapy approaches. Additionally, accurate immune response activation and immune suppression relief depend on an understanding of the immunotherapy’s controllable time and space. Thus, there is a great potential for therapeutic efficacy when tumor immunotherapy is combined with nanotechnology. In addition, significant consideration should be given to large-scale, reproducible commercial batches of nanomedicine formulations with improved efficacy and decreased toxicity. To achieve quick clinical conversion, a number of important factors need to be taken into account. First, by controlling the tumor microenvironment and metabolism, future research on metabolic pathways and cancer should be focused on enhancing cancer immunotherapy. Second, in order to specifically respond to the tumor microenvironment, nano-delivery devices with imaging capabilities and stimulus activation properties must be developed. Notably, tumor immunotherapy should be integrated into the development of nanomedicine’s diagnosis and treatment plans.

Third, interdisciplinary research is required to find new immunological targets and pathways that will make it easier to create new drug delivery methods. This research should involve experts from pharmacology, materials science, immunology, and other domains. As a whole, the development of nanomedicine systems should concentrate on both commercialization and their ability to reach the clinical context.

## Author contributions

All authors contributed equally to the writing of this manuscript. All authors provided critical feedback and helped shape the manuscript. All authors contributed to the article and approved the submitted version.

## Funding

This research is co-financed by the ERAPEDMED/PMT-LC grant —Personalized multimodal therapies for the treatment of lung cancer (ERAPERMED2020-342) funded by the European network grant and the Greek General Secretariat for Research and Innovation (GSRT).

## Acknowledgments

The author would like to thank Professor Konstantinos Zarogoulidis for reading the manuscript and providing useful comments and advice.

## Conflict of interest

The authors declare that the research was conducted in the absence of any commercial or financial relationships that could be construed as a potential conflict of interest.

## Publisher’s note

All claims expressed in this article are solely those of the authors and do not necessarily represent those of their affiliated organizations, or those of the publisher, the editors and the reviewers. Any product that may be evaluated in this article, or claim that may be made by its manufacturer, is not guaranteed or endorsed by the publisher.
